# The transport of liquids in softwood: timber as a model porous medium

**DOI:** 10.1038/s41598-019-55811-6

**Published:** 2019-12-30

**Authors:** H. C. Burridge, G. Wu, T. Reynolds, D. U. Shah, R. Johnston, O. A. Scherman, M. H. Ramage, P. F. Linden

**Affiliations:** 1 Department of Civil and Environmental Engineering, Skempton Building, Imperial College London, London, SW7 2AZ UK; 2 Melville Laboratory for Polymer Synthesis, Department of Chemistry, University of Cambridge, Lensfield Road, Cambridge, CB2 1EW UK; 3 Institute for Infrastructure and Environment, School of Engineering, University of Edinburgh, Edinburgh, EH9 3FG UK; 4 Department of Architecture, University of Cambridge, Cambridge, CB2 1PX UK; 5 Materials Research Centre, College of Engineering, Swansea University, Swansea, SA1 8EN UK; 6 Department of Applied Mathematics and Theoretical Physics, University of Cambridge, Centre for Mathematical Sciences, Wilberforce Road, Cambridge, CB3 0WA UK

**Keywords:** Engineering, Civil engineering, Fluid dynamics

## Abstract

Timber is the only widely used construction material we can grow. The wood from which it comes has evolved to provide structural support for the tree and to act as a conduit for fluid flow. These flow paths are crucial for engineers to exploit the full potential of timber, by allowing impregnation with liquids that modify the properties or resilience of this natural material. Accurately predicting the transport of these liquids enables more efficient industrial timber treatment processes to be developed, thereby extending the scope to use this sustainable construction material; moreover, it is of fundamental scientific value — as a fluid flow within a natural porous medium. Both structural and transport properties of wood depend on its micro-structure but, while a substantial body of research relates the structural performance of wood to its detailed architecture, no such knowledge exists for the transport properties. We present a model, based on increasingly refined geometric parameters, that accurately predicts the time-dependent ingress of liquids within softwood timber, thereby addressing this long-standing scientific challenge. Moreover, we show that for the minimalistic parameterisation the model predicts ingress with a square-root-of-time behaviour. However, experimental data show a potentially significant departure from this $\sqrt{{\boldsymbol{t}}}$ behaviour — a departure which is successfully predicted by our more advanced parametrisation. Our parameterisation of the timber microstructure was informed by computed tomographic measurements; model predictions were validated by comparison with experimental data. We show that accurate predictions require statistical representation of the variability in the timber pore space. The collapse of our dimensionless experimental data demonstrates clear potential for our results to be up-scaled to industrial treatment processes.

## Introduction

## Overview and Context

Timber is the only widely used building material that is genuinely sustainable inasmuch as the lifespan of timber used in construction typically exceeds the time taken to grow the raw material^1^. Greater use of timber as a building material offers significant benefits, including the potential for more sustainable urban developments and, by providing a meaningful sink of carbon, a role in alleviating anthropogenic greenhouse gas emissions^2^. Liquid transport within timber is of direct relevance to the chemical treatment of wood^3^, the preservation of archaeological timbers^4^, and the impregnation of timber to alter its mechanical properties^5,6^. The recent works by Song *et al*.^7^ and Frey *et al*.^8^ demonstrate that effective removal of lignin, by exposing the microstructure to aqueous chemical solutions, and its densification result in a modified wood-based material with unprecedented mechanical performance. In order to industrialise the lignin removal process, and benefit from this modified material, accurate prediction of the transport of liquids within the timber is needed. Our study provides the required insights and we validate a model which enables such predictions. Through simplified representations of the microstructure of softwood timber we accurately predict the transport of liquid treatments, thereby providing a significant step towards the more efficient and effective treatment of timber.

Pore space within widely used softwoods constitutes a significant portion of the timber volume (typically around 70%). Predicting the liquid transport within this space is challenging due to the the effects of interfacial tension and the wide distribution of pore sizes^9^ – considerations that are relevant to the liquid transport in all naturally occurring porous media. Consideration of the flows within bundles of capillary-tubes continues to yield theoretical advances^10^, but applying these advances to naturally occurring porous media remains challenging^11^. We demonstrate that softwood timber provides a model for natural porous media, with which to further scientific understanding.

Unlike other less complex porous media, e.g. Gruener & Huber^12^, the ingress of liquid within timber is not predicted accurately by application of Darcy’s law^13^ nor the classical Lucas–Washburn equation^14,15^, i.e. the ingress does not adhere to a $\sqrt{t}$ behaviour^9,16–18^. Simple modified models^16^ are in current use^19^, but these models lack a sound physical basis and the match between predictions and experimental data is poor. Petty’s model^20^ for gaseous transport within softwood timber, which considers the pore space to consist of long thin cylindrical voids within tracheids adjoined by small circular openings (which constitute a simplified model of bordered pits) is an exception. He compares predictions from the model with ‘smoothed’ data from experiments that show good agreement for the variation of the resistance to flow through a timber specimen with imposed pressure. The first model we develop, our ‘single-wood’ model, builds on Petty’s model^20^ by incorporating the effects of interfacial tension.

The seasonal growth of trees has resulted in characterisation of wood in two phases: early-wood (of larger cellular structure) and late-wood (of smaller pore size). Zillig *et al*.^21^ present a model that attempts to account for differences in the flow rates in these two phases but they do not accurately predict liquid uptake. Krabbenhoft & Damkilde^22^ account for the effect of these two wood phases at a macroscopic level in their double porosity model — a parameterisation which requires tuning to experimental data to provide good predictions of the liquid transport. Recent studies examine the effects of polar or non-polar liquids^18^ but Zillig^23^ concludes “that in modelling of liquid transport, the cellular level needs to be taken into account”. The research presented in this paper describes a model which successfully addresses this problem and will enable industrial processes, required to better exploit timber as an engineering material, to be improved.

## Results

**Figure 1:**
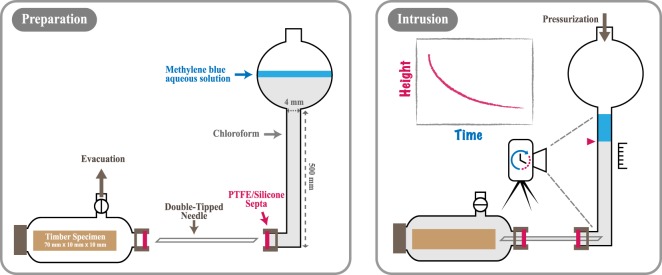
Schematic illustrations of the experimental set-up, both in a state prepared ready for experimentation (left-hand pane) and during experimentation as the chloroform intrudes the timber specimen (right-hand pane).

We compare the results of experiments that measure the liquid uptake by softwood timber specimens as a function of time to model predictions. Our experiments examined the uptake of chloroform by three specimens of Sitka spruce (Picea sitchensis), each of length (along the grain) ${L}_{0}\approx 70\,{\rm{mm}}$ and the cross-sectional area ${A}_{0}\approx 10\,{\rm{mm}}\times 10\,{\rm{mm}}$. In each of the twelve experiments conducted, a specimen was dried, subjected to a vacuum and then submerged in a reservoir of chloroform. The reservoir was connected to a high pressure line via a vertically aligned precision bore glass tube (Fig. 1). The volume of chloroform within the reservoir was selected such that the volume uptake could be recorded by measuring the height of the column of chloroform within the precision bore glass tube. Figure 1 provides illustrations of the set-up and the procedure: full details are provided within the Methods section.

### Modelling assumptions

Our model, a modification of the Lucus–Washburn equation^14,15^, describes the intrusion of a viscous fluid, predominately driven by imposed pressure gradients and further accounting for the effects of surface tension, within softwood (gymnosperm) timber. The (approximately) uni-directional alignment of the tracheid tubes within softwood timbers results in the dominant transport occurring along the grain, with the permeability in this direction being typically greater than that in the cross-grain directions by factors in the range 10^4^–10^6^, see Comstock^24^. We make the simplifying assumptions that timber is orthotropic and is impermeable in the cross grain directions. We further assume that the intruding flow is a creeping flow, in which inertial effects are insignificant: in our experiments the Reynolds numbers of the flow within the pore space were typically of order 10^−1^. Moreover, we assume that the intruding fluid causes no swelling of the timber micro-structure: in our experiments we selected chloroform as the intruding fluid, which has been shown to result in negligible swelling of softwood timber (see Methods section).

**Figure 2:**
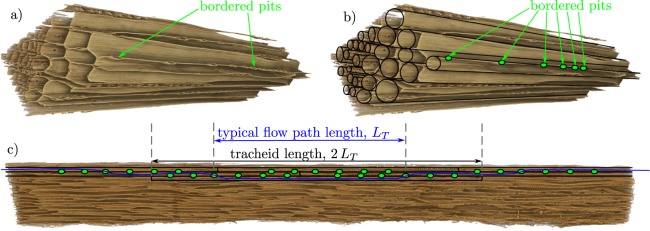
(**a**) Micro CT image of a section (approximately 0.6 mm in length) of specimen 3; (**b**) the same image with an overlaid illustration showing aspects of the statistical-wood model of cylindrical pore space (of varied radii) adjoined by small openings representative of the bordered pits; (**c**) micro CT image showing a longitudinal cross-section through specimen 3 (of approximately 6.4 mm in length) with an overlaid illustration showing aspects of the model, including an example flow path (blue line) illustrating that the flow path length within a tracheid is modelled as approximately half the physical length of the tracheid. For details see the Methods section.

We define the accessible porosity $\phi$ of the wood as the proportion of void space that is accessible to fluid flow (e.g. determined by helium pycnometry^25^). Guided by our understanding of the microstructure of softwood timbers^26^, we consider the pore-space to consist of relatively long thin tubes (the liquid transmitting tracheid lumena), the cross-sections of which do not vary significantly along their length. The effective radius of a tracheid lumen is denoted *r*_*T*_ and the characteristic length of the flow path within each tracheid *L*_*T*_ is taken to be half the physical length of the tracheid, see Fig. 2 for an illustration and Lancashire & Ennos^27^ for a detailed discussion. We envisage that the intruding flow advances through a series of tracheids with each connected to the next by a number *n*_*p*_ of small openings, representative of the bordered pits between adjoining tracheids. For simplicity, we model the losses through the bordered pits by consideration of the losses through small circular holes (as has previously been considered for gaseous flows in timber^20^) with the effective radius *a*_*p*_. See Methods section for a detailed discussion of the bordered pits.

The geometry of the pore space is determined by *r*_*T*_, *L*_*T*_, *n*_*p*_ and *a*_*p*_. First, we assume each parameter takes a single value throughout each specimen. However, seasonal growth results in substantial differences in the characteristic pore sizes found in early-wood and late-wood so a second ‘two-wood’ model is developed in which each geometric parameter takes one of two values representative of either early- or late-wood. Finally, a ‘statistical-wood’ model is developed which again consists of early- and late-wood phases and further accounts for the natural variations of the pore space within each phase. Each model geometry was informed by micro computed tomographic (CT) imaging of the timber specimens (see Methods section for details). Example images and an illustration of our statistical-wood model are provided in Fig. 2.

### Model

The intruding flow is driven by the total pressure difference Δ*P*, with contributions from both the imposed pressure difference Δ*P*_*a*_ and the effects of surface tension Δ*P*_*c*_ included. For our experiments $\Delta {P}_{a}={P}_{H}+{P}_{hyd}-{P}_{in}$, where $\Delta {P}_{hyd}$ denotes the hydrostatic pressure variation within the (vertical) liquid column used to determine the intruded volume (see Methods section), *P*_*H*_ denotes the pressure applied by the high pressure line at the upper surface of the liquid column (${P}_{H}=1.0\,{\rm{bar}}$ or 1.5 bar in our experiments) and *P*_*in*_ is the pressure within the air in the pore space beyond the front of the intruding fluid. The height of the liquid column $h=h(t)$ and, therefore, ${P}_{hyd}(t)=\rho \,g\,h(t)$, varies in time (where $\rho$ is the density of the intruding fluid and *g* the acceleration due to gravity). Values of *h* were deduced from the experimental images although, for our experiments, *P*_*H*_ was the dominant driving pressure with, typically, ${P}_{hyd} < 0.05\,{P}_{H}$. During the experiments, the air within the pore space (initially at a pressure of ${P}_{in}(0)=0.001$ bar, achieved via exposure to a vacuum for a sufficiently long period, see Methods section for full details) was forced to occupy an increasingly small volume as the intruding liquid advanced within the submerged specimen from both ends, and so the pressure of the air beyond the intruding front also varied with time. Assuming that air behaves as an ideal gas, the pressure beyond the fronts of the intruding fluid is ${P}_{in}(t)={P}_{in}(0)\,{L}_{0}/({L}_{0}-2\,{L}_{f}(t))$, where ${L}_{f}(t)$ is the length of the intruding front (since in our experiments fluid is forced into the submerged specimen from both ends, the expression for ${P}_{in}(t)$ includes a factor of two).

In the presence of surface tension at the front of the intruding fluid the small pore size makes capillary forces potentially significant. The precise role of the capillary forces as liquids intrude along the tracheids and pass through the adjoining bordered pits are complicated by any roughness at the tracheid wall and by the complex geometry of the bordered pits, e.g. the pit borders and pit membrane including the torus and margo fibres therein (see Methods section and images within Siau^26^ for further details). The metastable transport of water in tree implies that there must be a lack of nucleation sites along the transport paths^28^ and hence that the tracheids are relatively smooth. The capillary forces as the fluid front passes through a bordered pit are potentially significant since the effective radius of the pits is ${a}_{p}\approx 0.2$ *μ*m (see Methods section). The integral effect of these capillary forces as they draw liquids along the transport pathways will approximately scale with the length (in the streamwise coordinate) divided by the radius. Hence we expect the capillary forces as the flow intrudes along the tracheids (${L}_{T}/{r}_{T}\approx 80$) to be at least two orders of magnitude larger than those as the front passes through the bordered pits (${L}_{p}/{a}_{p}\approx 0.5$, where ${L}_{p}\approx 0.1$ *μ*m is the length of the pits in the streamwise coordinate, see images in Siau^26^). Hence, we neglect the capillary forces as the flow passes through the bordered pits and we include capillary forces via the approximation that the effective change in pressure is given by $\Delta {P}_{c}=(2\gamma \,\cos \,\theta )/{r}_{T}$, where *γ* is surface tension at the air-liquid interface and *θ* is the contact angle. The total driving pressure difference along any particular pathway of tracheids can therefore be written as

1 $$\Delta P=\Delta P({r}_{T},t)={P}_{H}+{P}_{hyd}(t)+\Delta {P}_{c}({r}_{T})-{P}_{in}(t).$$

For a given liquid volume flow rate *q*_*T*_ along the flow path within a tracheid, Poiseuille flow implies that the pressure drop over the length *L*_*T*_ of the tracheid as

2 $$\Delta {P}_{w}=\frac{8\mu {L}_{T}{q}_{T}}{\pi {r}_{T}^{4}},$$

where *μ* is the dynamic viscosity of the intruding fluid. Poiseuille flow is known to be valid for laminar flows along smooth cylindrical vessels; however, flows along rough walled vessels of more complex cross-section geometry can be accounted for by taking an effective radius (e.g. Herwig *et al*.^29^), an approximation made herein. The pressure drop across a bordered pit Δ*P*_*pit*_ is determined by assuming it is a small circular hole. Sampson^30^ derived an expression for the pressure drop in a creeping flow through a small circular hole, of radius *a*, within an infinite plate which is $3\mu q/{a}^{3}$ (see also Roscoe^31^), where *q* denotes the volume flow rate. Taking the tracheid radius to be much larger than the effective radius of the bordered pit makes dimensional considerations of a creeping flow through a small circular hole within a finite sized plate, of radius *r*, relevant and these imply that the pressure drop is of the form

3 $$\Delta {P}_{pit}=\frac{3\mu q}{{a}^{3}}(1-\frac{{a}^{m}}{{r}^{m}}),$$

where the exponent *m* is unknown. On physical grounds, we expect this pressure drop to vary with the area of the tracheid end area and therefore set $m=2$, as has been shown to be appropriate^32^ but since $a/r$ is of order 10^−2^ the precise choice of *m* is of no practical consequence. Each tracheid is adjoined to its neighbour by *n*_*p*_ bordered pits and, as the pressure drop through each pit follows (3), we can write the pressure drop over the length of the flow path within a tracheid and on entering the adjoining tracheid as $\Delta {P}_{T}=\Delta {P}_{w}+\Delta {P}_{pit}/{n}_{p}$. Hence, the mean pressure gradient is

4 $$\delta {P}_{T}=\frac{\Delta {P}_{T}}{{L}_{T}}=\frac{8\mu {q}_{T}}{\pi {r}_{T}^{4}}+\frac{3\mu {q}_{T}}{{n}_{p}{a}_{p}^{3}{L}_{T}}(1-\frac{{a}_{p}^{2}}{{r}_{T}^{2}}).$$

The total pressure drop over the length ${L}_{f}(t)$ of the creeping intruding flow at any instant is equal to the total driving pressure difference Δ*P*. Integrating the pressure gradient (4) over ${L}_{f}(t)$ to recover the total pressure loss within the intruding flow and setting this equal to the total driving pressure difference gives

5 $$\Delta P={\int }_{0}^{{L}_{f}(t)}\,\delta {P}_{T}\,{\rm{d}}l=\mu \,{q}_{T}[\frac{8}{\pi {r}_{T}^{4}}+\frac{3}{{n}_{p}{a}_{p}^{3}{L}_{T}}(1-\frac{{a}_{p}^{2}}{{r}_{T}^{2}})]{L}_{f}(t).$$

Noting ${q}_{T}=\pi {r}_{T}^{2}\frac{d{L}_{f}(t)}{dt}$ and writing

6 $${B}_{T}^{-1}=\frac{8}{\pi {r}_{T}^{4}}+\frac{3}{{n}_{p}{a}_{p}^{3}{L}_{T}}(1-\frac{{a}_{p}^{2}}{{r}_{T}^{2}}),$$

gives

7 $$\frac{\Delta P}{{L}_{f}(t)}=\frac{\mu }{{B}_{T}}\pi {r}_{T}^{2}\frac{{\rm{d}}{L}_{f}(t)}{{\rm{d}}t}.$$

With the initial condition that ${L}_{f}(0)=0$, we obtain

8 $${L}_{f}={[\frac{2{B}_{T}}{\mu \pi {r}_{T}^{2}}{\int }_{0}^{t}\Delta P({r}_{T},t){\rm{d}}t]}^{1/2}.$$

In our single-wood model the parameters determining the cellular structure of the wood each take a single value and the length of fluid fronts intruding both ends of the specimen evolve according to (8). A prediction of the intruded volume of fluid can therefore be determined by numerical solution of the initial value problem

9 $$V(t)=2\phi {A}_{0}{L}_{f}=2\phi {A}_{0}{[\frac{2{B}_{T}}{\mu \pi {r}_{T}^{2}}{\int }_{0}^{t}\Delta P{\rm{d}}t]}^{1/2},$$

with initial conditions $V(0)=0$, ${P}_{hyd}(0)=\rho g{h}_{0}$, and ${P}_{in}(0)=0.001\,{\rm{bar}}$.

In order to reflect the typical description of timber in terms of early- and late-wood phases we further consider a two-wood model of timber. The geometric parameters in each take one of two (different) values. We denote the volume fraction of the early-wood by *α* and use the subscripts ‘*e*, *l*’ to denote parameters corresponding to the early- and late-wood phases, respectively. Again predictions of the intruded volume of fluid can be determined by numerical solution of

10 $$V(t)=2{A}_{0}[{\phi }_{e}\,\alpha {(\frac{2{B}_{e}}{\mu \pi {r}_{e}^{2}}{\int }_{0}^{t}\Delta {P}_{e}{\rm{d}}t)}^{1/2}+{\phi }_{l}\,(1-\alpha ){(\frac{2{B}_{l}}{\mu \pi {r}_{l}^{2}}{\int }_{0}^{t}\Delta {P}_{l}{\rm{d}}t)}^{1/2}],$$

with the same initial conditions as above.

**Figure 3:**
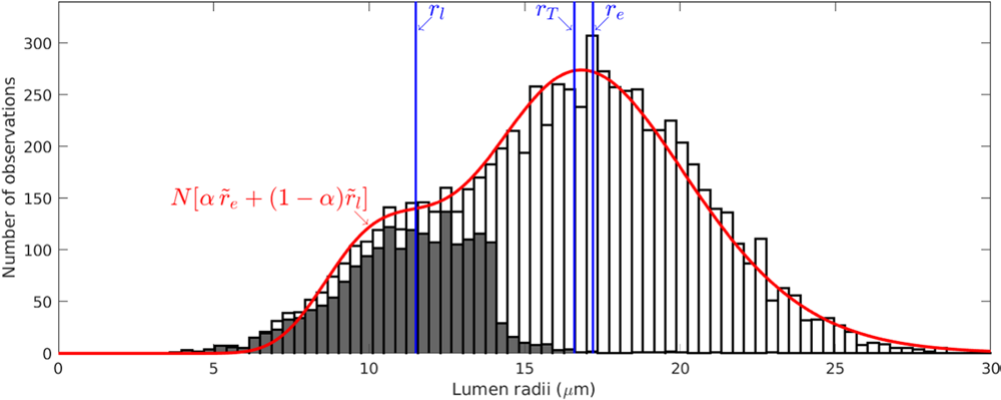
Histogram of the lumen radii in specimen 2, determined by the micro-CT measurements reported in Reynolds *et al*.^34^; cells therein determined to be within the late-wood phase are highlighted in grey. Blue vertical lines mark the mean radius *r*_*T*_ for the single-wood model and the mean radii of the late- and early-wood phases used in the two-wood model. The red curve illustrates the distribution of radii used in the statistical-wood model, where *N* is the total number of observations.

Finally, we consider a statistical-wood model that accounts for the variations in small-scale geometry that arise during the growth of trees by assuming that the geometric properties of the pore space in both the early- and late-wood phases each follow a different log-normal distribution. For geometric parameters, which must take strictly positive values, log-normal distributions are both common in nature and expected in statistics under either the central limit theorem or maximal entropy considerations^33^. Moreover, Fig. 3 shows that these log-normal distributions fit well the data from micro-CT measurements, see Reynolds *et al*.^34^ and their Fig. 9 for further details. We imposed this statistical distribution by introducing the probability function *p*_*i*_ which is log-normally distributed with a mean of unity and a standard deviation *σ*. The standard deviations are determined by the data from the CT scans of the specimens, reported in Table 1. We write ${\tilde{l}}_{T}={p}_{i}{l}_{T}$, ${\tilde{r}}_{T}={p}_{i}{r}_{T}$, ${\tilde{n}}_{p}={p}_{i}{n}_{p}$, ${\tilde{a}}_{p}={p}_{i}{a}_{p}$,

11 $${\tilde{B}}_{T}^{-1}=\frac{8}{\pi {\tilde{r}}_{T}^{4}}+\frac{3}{{\tilde{n}}_{p}{\tilde{a}}_{p}^{3}{\tilde{l}}_{T}}(1-\frac{{\tilde{a}}_{p}^{2}}{{\tilde{r}}_{T}^{2}}),$$

and

12 $$\Delta \tilde{P}={P}_{H}+{P}_{hyd}(t)+\frac{2\gamma \,\cos \,\theta }{{\tilde{r}}_{T}}-[{P}_{in}(0)\frac{{L}_{0}}{{L}_{0}-2\,{\tilde{L}}_{f}(t)}],$$

where ${\tilde{L}}_{f}(t)$ is the distribution of the lengths of the intruding fronts at any instant,

13 $${\mathop{V}\limits^{ \sim }}_{e}(t)=2\,{\int }_{0}^{{\phi }_{e}\alpha {A}_{0}}\,{\int }_{0}^{\infty }\,{[\frac{2{\tilde{B}}_{e}}{\mu \pi {\tilde{r}}_{e}^{2}}{\int }_{0}^{t}\Delta \tilde{P}{\rm{d}}t]}^{1/2}\,{\rm{d}}{p}_{i}\,{\rm{d}}A,$$

and an equivalent expression evaluated for ${\mathop{V}\limits^{ \sim }}_{l}(t)$. Within our statistical-wood model, i.e. $\mathop{V}\limits^{ \sim }(t)={\mathop{V}\limits^{ \sim }}_{e}(t)+{\mathop{V}\limits^{ \sim }}_{l}(t)$, the effective distribution of each geometric parameter is taken to be the sum of two log-normal distributions, with means and variances that vary between early- and late-wood. For example, the effective distribution of $\tilde{r}=\alpha \,{\tilde{r}}_{e}+(1-\alpha ){\tilde{r}}_{l}$ is shown in Fig. 3 and exhibits a good approximation to the data measured by micro-CT scans.

**Table 1:** The characteristic geometries obtained from the micro-CT imaging (see^34^ for full details) that are used as the input parameters for the model.

	Entire specimen	Early-wood phase	Late-wood phase
**Specimen 1**			
Proportion of volume	1	*α* = 0.83	(1 − *α*) = 0.17
Measured porosity	0.73	0.75	0.63
Tracheid radius	*r*_*T*_ = 15.8 *μ*m	*r*_*e*_ = 16.6 ± 3.3 *μ*m	*r*_*l*_ = 11.5 ± 3.1 *μ*m
**Specimen 2**			
Proportion of volume	1	*α* = 0.88	(1 − *α*) = 0.12
Measured porosity	0.75	0.76	0.63
Tracheid radius	*r*_*T*_ = 16.6 *μ*m	*r*_*e*_ = 17.2 ± 2.9 *μ*m	*r*_*l*_ = 11.5 ± 2.4 *μ*m
**Specimen 3**			
Proportion of volume	1	*α* = 0.89	(1 − *α*) = 0.11
Measured porosity	0.75	0.76	0.63
Tracheid radius	*r*_*T*_ = 16.7 *μ*m	*r*_*e*_ = 17.1 ± 2.7 *μ*m	*r*_*l*_ = 11.8 ± 2.0 *μ*m

Where provided, the tolerances indicate the standard deviation obtained and input into the statistical-wood model.

### Validation and insights

The models were parametrised by reference data for the fluid properties and CT measurements of each specimen pore space geometry; the only exception being the effective size of the bordered pits which remained the single free parameter in fitting model predictions to experimental data. The values of *a*_*p*_ determined from the fitting agree well with values reported in the literature and we show that the losses due to the bordered pits implied by our models are in-line with experimental measurements reported in other studies (see Methods section). Moreover, our results show that the model predictions accurately reflect changes in experimental conditions without need to change the parameterisation of the bordered pits.

**Figure 4:**
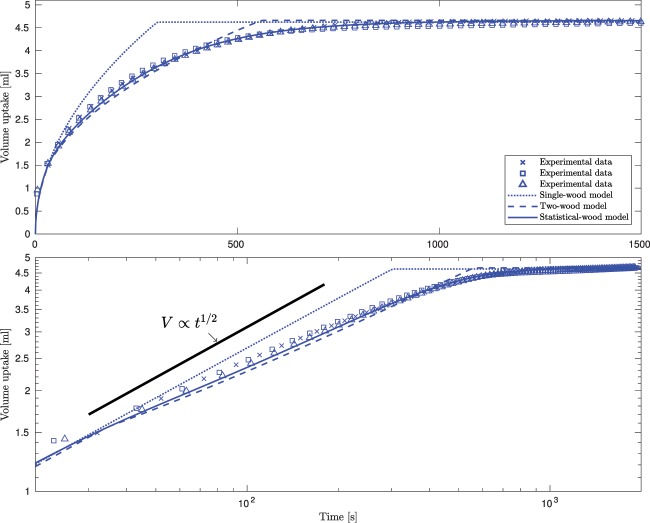
Data for specimen 2 (upper pane: linear axes, lower pane: log-log axes). The experimental data (blue markers) are from three notionally identical independent experiments and demonstrate the degree of repeatability between experiments. The results of the single-wood and two-wood models of timber are marked by the blue dotted and blue dashed curves respectively. The solid blue curve shows the predictions of the statistical-wood model.

In order to compare the model predictions with experiments we present data on both linear and logarithmic axes. Figure 4 shows the results of three notionally identical experiments carried out on specimen 2; the data illustrate that these experiments are repeatable with variations between experiments less than 3%. The experimental data show a clear departure from the $V\propto {t}^{1/2}$ expected from either Darcy’s law or the Lucas–Washburn equation. The lower-pane of Fig. 4 shows that our single-wood model adheres to the $V\propto {t}^{\mathrm{1/2}}$ behaviour throughout the filling process, leading to difference of up to 25% between predictions and the measured volume uptake. Both our two-wood and statistical-wood models exhibit a departure from the $V\propto {t}^{\mathrm{1/2}}$ behaviour, with the two-wood model able to predict the volume uptake to within 10%. However, in order to obtain quantitative agreement the statistical properties of the pore space within the timber needs to be included. Using the values obtained from the micro-CT scans the agreement between the predictions of the statistical-wood model and the experimental data is within 3% at all times, which is within the bounds of experimental uncertainty. This agreement demonstrates the ability of our statistical-wood model to predict the time dependent uptake of liquid by timber with an accuracy not previously reported.

**Figure 5:**
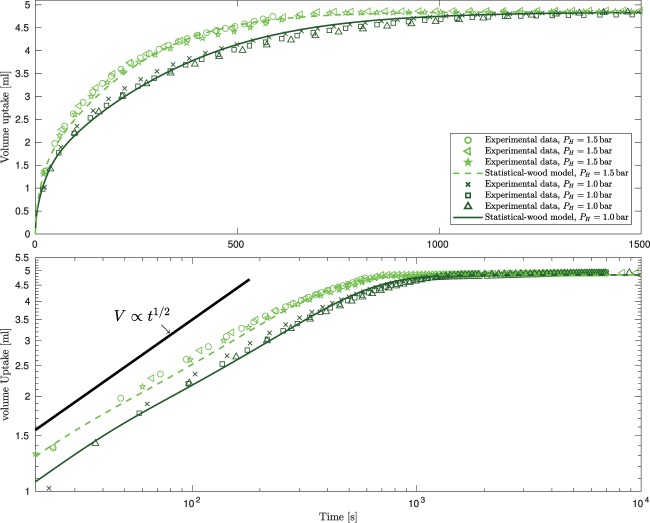
Data from six independent experiments on specimen 3 at two different imposed pressures: dark-green markers denote data for ${P}_{H}=1.0\,{\rm{bar}}$ and light-green markers ${P}_{H}=1.5\,{\rm{bar}}$. The predictions of the statistical-wood model at these two imposed pressures are marked by the solid dark-green and dashed light-green curves, respectively. Upper pane: the data on linear axes, lower pane: the data on log-log axes.

**Figure 6:**
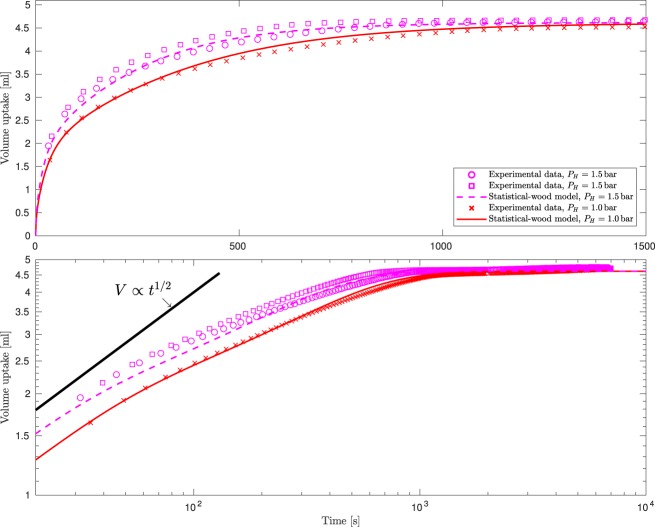
Data from three independent experiments on specimen 1: red markers denote data for ${P}_{H}=1.0\,{\rm{bar}}$ and magenta markers ${P}_{H}=1.5\,{\rm{bar}}$. The predictions of the statistical-wood model at these two imposed pressures are marked by the solid red and dashed magenta curves, respectively. Upper pane: the data on linear axes, lower pane: the data on log-log axes.

Figures 5 and 6 show the results of the nine experiments carried out on specimens 3 and 1, respectively. These experiments were carried out at two different imposed pressures, ${P}_{H}=1.0$ and 1.5 bar. The maximum difference between otherwise identical experiments is typically less than 4% and never above 6% demonstrating good repeatability across a range of timber specimens. The discrepancy between the predicted and observed uptake never exceeds 5%, demonstrating that without altering any of the geometrical parameters the model predictions accurately respond to changes in experimental conditions (in this case a different imposed pressure).

The scale of the observed departure from the $V\propto {t}^{\mathrm{1/2}}$ behaviour depends on the proportion of the late-wood phase. For example, the departure is most stark for specimen 1 which contained the greatest proportion of late-wood, see Table 1 (compare the lower-pane of Fig. 6 with those of Figs. 4 and 5).

A scaling analysis of the data (see Methods section) highlights that in our experiments the liquid transport was dominated by the imposed pressure difference $\Delta {P}_{a}={P}_{H}+{P}_{hyd}(t)-{P}_{in}(t)$ with the effects of surface tension accounting for only between 7% and 11% of the total liquid uptake. To achieve good quantitative agreement with the time-dependent uptake data, it is necessary to include the effects of surface tension even though these effects did not dominate the transport (despite the fact that the initial (maximum) imposed pressure differences were relatively modest, $1.0\,{\rm{bar}}\le \Delta {P}_{a}(0)\le 1.6\,{\rm{bar}}$, in our experiments).

**Figure 7:**
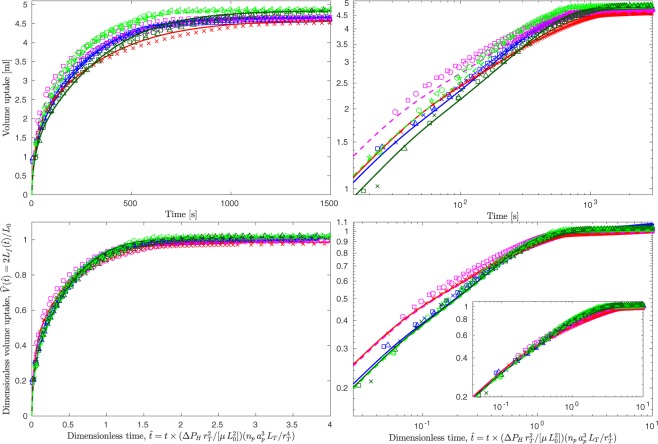
The top two panes show the data from all twelve experiments and the five model predictions, with all of the data marked as per Figs. 4, 5 and 6 (see legends therein for details). The lower two panes show the same data scaled by the volume of the pore space within the specimens and the time scale $(\mu \,{L}_{0}^{2}/[\Delta {P}_{H}\,{r}_{T}^{2}])\,({r}_{T}^{4}/[{n}_{p}\,{a}_{p}^{3}\,{L}_{T}])$. The data on the primary axes of the lower two panes exhibit a good collapse when the geometrical parameters within the time scale take values appropriate for the early-wood phase. The data on the axis inset within the lower right-hand pane shows a good collapse can be achieved at early time when the geometrical parameters within the time scale take the (volume) weighted mean of values appropriate for the early- and late-wood phases.

The upper two panes of Fig. 7 show the data from all twelve experiments replotted on the same axes. These highlight differences in the liquid uptake between the three different timber specimens at the same imposed pressure (blue, dark-green and red symbols), e.g. variations of around 16% are evident at $t\approx 700\,{\rm{s}}$. The data for the higher imposed pressure, ${P}_{H}=1.5\,{\rm{bar}}$ (light-green and magenta data), shows the liquid uptake is markedly faster. In the dimensionless form identified by our scaling analysis, the data for a given specimen at differing imposed pressure exhibits a near perfect collapse — see the agreement between the red and magenta data, and between the dark- and light-green data in the lower two panes of Fig. 7. With this dimensionless presentation of the data, with time scaled by $T=(\mu \,{L}_{0}^{2}/[\Delta {P}_{H}\,{r}_{T}^{2}])\,({r}_{T}^{4}/[{n}_{p}\,{a}_{p}^{3}\,{L}_{T}])$, and taking values for the geometric parameters representative of early wood (constituting the bulk of the specimen volumes), the differences in the liquid uptake between the three specimens are also significantly reduced (e.g. the variations decrease to around 5% at dimensionless times $\hat{t}\approx 1.5$, equivalent to $t\approx 700\,{\rm{s}}$). Differences between the specimens still occur at relatively small dimensionless times (see the data plotted on the main axes of the bottom right-hand pane of Fig. 7); however, for $\hat{t}\ge 0.5$, ($t\gtrsim 230\,{\rm{s}}$) the data from all three specimens collapse indicating that this scaling provides predictions of use for the industrial treatment of timber. Moreover, scaling the data with the time scale *T* but taking the geometric parameters based on the (volume) weighted mean of the values representative of early- and late-wood within each specimen provides a good collapse for small dimensionless times — the data scaled in this way is presented on the axes inset within the lower right-hand pane of figure. This indicates that for small dimensionless times the liquid transport in both early- and late-wood is important but for $\hat{t}\ge 0.5$ the transport in the early-wood phase dominates.

In scaling the data we assumed that the dominant force balance was between the driving pressure and the viscous stresses as the flow passes through the bordered pits — the good collapse of the data suggests that the drag associated with flow through the bordered pits controls the rate of liquid transport in softwood timber. This finding is further supported by the good agreement between the losses through the bordered pits implied by our data, with that published in the existing literature (see Methods section).

## Conclusions and Implications

We have developed simplified models of softwood pore space using micro-CT measurements. The models enable analytical descriptions of the viscous and interfacial interactions between the cellular structure and an intruding liquid. The models provide predictions of the liquid uptake as a function of time; comparison to experimental data highlights that, when the natural variation of the pore space geometry is accounted for, the predictions capture the complex time-dependent behaviour of the uptake accurately and precisely — a feat which has remained an outstanding challenge in fundamental wood science^9^. The agreement between experimental and modelled data provides confidence in our finding that in the specimens tested the resistance to flow through the bordered pits was larger than that at the tracheid lumen wall by a factor of order 10^3^, and our findings indicate that even in green-wood timber that factor might remain as large as 200–400. This highlights a research challenge, namely to resolve the dynamics of the flow through the complex geometry of the bordered pits (perhaps requiring better understanding of their biochemistry) in both dried softwood timber and, furthermore, in the trees that produce it.

Timber is the only widely used construction material we can grow^1^ and its increased usage in construction offers potentially profound benefits^2^. The ability of our model to predict liquid transport within timber offers opportunities to extend the scope for the use of timber in modern construction. For example, the widespread practice of increasing the resilience of timber by impregnation with liquid preservative treatments could be informed by industrial-scale application of our model. Moreover, attempts to alter the properties of timber and enable its wider use in modern construction, that involve exposure of the micro-structure to liquid treatments are ongoing^5–8^. Our model is parameterised by data from CT scans made at resolutions achievable for industrial scale timber^35^ in combination with recently published correlations between these measurements and the timber micro-structure (measured in higher-resolution scans)^34^. It provides the accuracy required to predict the transport of these liquids within timber and determine the regions exposed to the desired liquid treatment. Scaling the data confirmed that a balance between the dominant driving pressure and the viscous losses due to the bordered pits controls the rate of liquid uptake in softwood timber — the success of this scaling enables our predictions of the liquid transport to be robustly up-scaled for relevant industrial applications.

Moreover, we have developed the ability to model the physical interactions between a fluid flow and a naturally occurring porous media analytically, and predict the uptake of an intruding liquid front using simple models. Continued research into the use of softwood timber as a model porous media could result in further meaningful contributions to understanding that might shed light on the fundamental and highly-relevant flows within more generic porous media. Finally, our findings have implications for the role of the bordered pits in the water transport within living trees, that warrant further investigation.

## Methods

We carried out experiments designed to enable repeatable measurements of the liquid volume within the pore space of oven-dried specimens of softwood timber. Measurements were taken, as a function of time, when liquid was forced to intrude the timber specimens under pressure in a manner which facilitated direct comparison with the predictions of our model. The experimental set-up was kept deliberately simple so as to enable the replication of our results in other laboratories.

### Experimental set-up

Three specimens of Sitka spruce (Picea sitchensis) were cut from kiln-dried knot-free timber supplied by BSW Timber Ltd (UK). Prior to commencing each experiment a specimen was selected, oven dried (in a BINDER, FD23 drying and heating oven) at $103.0\pm 0.3\,^\circ {\rm{C}}$ until its mass ceased to vary (approximately 12 hours) and its precise dimensions were measured using vernier callipers (to within ±0.01 mm). The mass (measured with a METTLER TOLEDO, AL204 analytical balance) and dimensions for a given specimen were notionally identical between experiments — indicating that irreversible changes in the biochemistry of the specimens was not caused by our experiments.

The specimen was then sealed inside a 10 ml Schlenk tube (customised to enable the specimen to be inserted and sealed within) and allowed to cool to room temperature at atmospheric pressure. The Schlenk tube was then partially submerged in a water bath (approximately 5 litres in volume) at room temperature to ensure that each experiment remained approximately isothermal throughout its duration. The two outlets of the Schlenk tube were position just above the water level. One of the outlets was screw threaded and sealed with a cap and PTFE/Silicone septa. The other (side-arm) outlet of the Schlenk tube was connected to a vacuum line and the tube’s (greased) stopcock valve opened to expose the specimen to a pressure of approximately 0.001 bar. Time was then allowed for the specimens to reach an equilibrium (over approximately 30 minutes) so that pore space within the specimen contained relatively low mass of air (at 0.001 bar). During this time, the intruding fluid (chloroform) was prepared within a customised volumetric apparatus.

Chloroform (analytical reagent grade) was selected, primarily as we could find no evidence of chemical interactions between softwood timber and chloroform, and the available data show negligible swelling of softwood timber in chloroform^36^. We note that there are no properties unique to chloroform that affect our modelling, and therefore expect our results to hold for liquids for which the chemical interactions between the timber and the liquid are not dominant in determining the transport. The volumetric apparatus (a 100 mL reservoir joined to a precision 4 mm bore tubing of length 500 mm) was aligned vertically and partially filled with chloroform (approximately 30 mL). The relatively volatile nature of chloroform ensured that it was easily removed from the specimen during oven drying post experimentation. However, this resulted in the need to ensure that evaporation at the air-chloroform interface was inhibited during experimentation. With chloroform being both of greater density $\rho =1.49$g/mL than, and immiscible with, water we inhibited the effects of evaporation by removing the air-chloroform interface through the introduction of a small volume (approximately 2 mL) of dyed water floating on top of the chloroform. As a dye we selected methylene blue (Fischer Scientific UK, >95% pure) which dissolves well in water but is not soluble in chloroform. Throughout our experiments the interface between the (transparent) chloroform and the (dark) methylene blue aqueous solution (0.1 mol/L) remained sharp and visually obvious. At the base of the precision bore tubing, the glassware was threaded and sealed with a screw-cap and a PTFE/Silicone septa (providing good chemical compatibility with chloroform). The left-hand pane of Fig. 1 shows a schematic illustration of the experimental set-up in the state described thus far, with the specimen prepared ready for experimentation.

A standard digital SLR camera (two differing makes and models were used during experimentation with no bias evident in results) was positioned to record images of the experiment. A ruler was hung beside the precision bore tubing to provide a scale of reference for the images. One image was recorded every two seconds with the chloroform-aqueous interface remaining clearly visible. To initiate the experiment, the septa at the end of the volumetric apparatus was pierced using a double-tipped needle (with deflected tips, gauge 20, Sigma-Aldrich) which then filled from one end with chloroform. Once filled the other end of the needle was used to pierce the septa sealing the Schlenk tube. Due to the pressure difference induced by the vacuum, the Schlenk tube then rapidly filled with chloroform, submerging the specimen in the chloroform and the Schlenk tube was then sealed from the vacuum line by closing the stopcock valve. The top of the volumetric apparatus was then connected to a high-pressure nitrogen (N_2_) line and the pressure controlled and monitored using a pressure regulator and gauge (WIKA, Model 612.20, NS63). For the majority of experiments the high-pressure line was maintained at a pressure of 1.5 bar (i.e. approximately 0.5 bar above atmospheric pressure). To test the models abilities to predict uptake behaviours at differing driving pressures some experiments were ran with the high-pressure line maintained at 1.0 bar (i.e. approximately atmospheric pressure).

Throughout the experiment the pressure difference between the high-pressure line and the air within the specimen pore space (initially at 0.001 bar) forced the chloroform to intrude the specimen. This continued until the air within the pore space was compressed such that its pressure was in balance with that applied by the high-pressure line — a process which took several hours. As chloroform intruded the specimen the chloroform-aqueous interface was drawn downwards within the precision bore tube. By comparing the height of the interface in any two images and knowing the cross-section area of the precision bore tube, the volume of chloroform that had intruded the specimen could be inferred. The right-hand pane of Fig. 1 shows a schematic illustration of the experimental set-up during experimentation.

Experiments were run until the chloroform-aqueous interface became stationary, which took between two and three hours. The specimen was then removed from the chloroform and quickly sealed within a plastic tube (of known mass). The sealed tube was then weighed so that the mass of the specimen and the chloroform (including any chloroform that evaporated subsequent to the specimens removal) could be simply deduced — deducting the mass of the specimen (measured just prior to experiment) provided a reliable estimate of the total mass of chloroform that intruded within the specimen during experimentation and dividing by the intruding fluid density provided the total intruded volume. From CT scans of the specimens^34^ the total proportion of void space within each specimen, i.e. porosity, was measured to be within the range 73–75%, see Table 1. At the end of each experiment the total fluid volume that had intruded within the specimen (deduced from measurements of the mass of chloroform) was found to be approximately 92% of the total pore volume. This is consistent with the findings of^25^ who (based on measurements of timber specimens taken from the same batch of Sitka spruce as the specimens reported herein) determined that approximately 92% of the total pore space was ‘accessible’, in their case either accessible to liquid monomers or helium pycnometry, i.e. some 8% of the pore space was inaccessible even to some of the smallest gas molecules. This is our justification in taking $\phi$ to be 92% of the measured porosity (see Table 1). Reassuringly, this indicates that, informed by the experiments of Wu *et al*.^25^, each of our experiments were run for a sufficiently long period of time that we achieved a pore filling ratio of 100% — see^25^ for a full discussion of ‘accessible pore space’ and ‘pore filling ratio’.

### Image analysis

The sharp nature of the interface and the stark contrast in the light absorption properties between the (transparent) chloroform below, and the (dark) methylene blue aqueous solution above, the interface provided that it was adequate to store and analyse the images recorded as grey-scale jpeg images (of 12 Mpix resolution). The stored images were analysed using Matlab, first cropping them to include only the pixel columns inside the, 4 mm wide, precision bore tube. These cropped images were then horizontally integrated to produce a single column of light intensities within the precision bore tube, from which the chloroform-aqueous interface could be readily identified by locating the maximum in the light intensity gradients. Identification of the location of the chloroform-aqueous interface was found to be robust to within 1 pixel, providing estimates of the intruded volume to an accuracy corresponding to approximately 0.004 mL (or around 0.1% of the total volume intruded). From both the position of the chloroform-aqueous interface and the time-stamps of the recorded images, combined with our measurements of the total intruded mass, we constructed time series of the intruded mass (or volume) for each experiment.

### A review of the micro-CT study of our timber specimens

Reynolds *et al*.^34^ scanned the present three timber specimens at two different resolutions. The entirety of all three specimens were scanned at a relatively low-resolution. From these scans the CT numbers obtained provided measurements of local bulk density within each specimen. The proportion of late- and early-wood within specimens could then be estimated (based on a threshold value, see^34^ for a full discussion) and the average porosity within each wood-phase was then calculated. Portions of the specimens were scanned at higher resolutions which enabled detailed characterisation of the pore space within the tracheids to be made. This could be correlated with the CT number measured at low-resolution in the same area to give estimates of the local porosity over the entire specimen. Additional high-resolution scans, over extended lengths of the specimens, provided estimates of the physical length of the tracheids. No statistically significant variation in the tracheid lengths was observed between the early- and late-wood phases. The length of the tracheids was found to be approximately 2.5 mm with a standard deviation of around 0.5 mm. By correlating measurements made at both low- and high-resolutions, data from the high-resolution scans yielded estimates for (all but one of) the geometric parameters required by our model — estimates which were obtained from measurements over the entirety of each specimen. Table 1 presents some of the parameters relevant to our model.

### Model parametrisation and losses through the modelled pits

To compare our model to experimental data we take 0.563mPa s for the viscosity of chloroform^37^, *γ* = 0.036 N/m for the surface tension at the chloroform-air interface^38^ and we take $\theta =0^\circ$ to be characteristic of the contact angle^38^. While our micro-CT study^34^ was carried out at resolutions for which estimates of the number of bordered pits per tracheid could be made, we were not able to determine the proportion of the pits which were aspirated, i.e. effectively closed to the intruding fluid. From our CT data, we estimate that on average the total number of bordered pits per tracheid was approximately 20, which agrees well with the value presented by Phillips^39^ who also examined kiln-dried softwood timber. Phillips^39^ went on to evaluate the proportion of aspirated pits as being approximately 95% in early-wood and around 65% in late-wood (see also^40^ for broadly similar values); following this we parametrise our model using ${n}_{e}=20\times 0.05=1$ and ${n}_{l}=20\times 0.35=7$ – these estimates agree well with those of other studies, for example^41^. The remaining parameters required by our models, with the exception of the effective size of the pit openings, are all determined quantitatively from our micro CT imaging of the timber specimens^34^ — a summary of which is presented in Table 1. Following Lancashire & Ennos^27^, in all our modelling we characterise the length of the flow path within each tracheid to be half of the physical length of the tracheid, i.e. ${L}_{T}=1.25\,{\rm{mm}}\pm 0.25\,{\rm{mm}}$.

The precise geometry of an unaspirated (i.e. open) bordered pit is complex, for example, see the scanning electron microscopy images and schematic illustrations in Siau^26^ and Usta & Hale^40^. As such, even with suitably high-resolution images it is not possible to rigorously determine the effective pit opening areas from the numerous tiny openings within the pit margo membrane. Siau^26^ suggests that for softwood timber the effective pit opening diameters lie in the range 0.02 *μ*m–4 *μ*m, noting that the logarithmic mean of the pit opening diameters is 0.3 *μ*m. Fitting our experimental data and model we find that pit openings of equivalent diameters $2{a}_{p}=\{0.41,0.47,0.43\}\,\mu m$ are appropriate for specimens 1, 2 and 3, respectively. We take these values to be characteristic of the pit openings in both early- and late-wood — these values all lie well within the range determined by Siau^26^ and are reassuringly close to the logarithmic mean reported therein.

Moreover, with these values our model enables the hydraulic resistivity of the pits ${R}_{p}=3\mu /({n}_{p}{a}_{p}^{3}{L}_{T})$ to be evaluated directly; both the early- and late-wood within the three specimens modelled fall within the range $14 < {R}_{p} < 150\,{\rm{MPa}}\,{\rm{s}}/{{\rm{mm}}}^{4}$. These values are somewhat higher than the values, $2\le {R}_{p}\le 20\,{\rm{MPa}}\,{\rm{s}}/{{\rm{mm}}}^{4}$, for the pit resistivity in softwood stems of the *Pinaceae* family reported by Pittermann *et al*.^42^. They inferred their values from measurements of the total resistivity of intact green-wood stems, combined with measurements and a simplified model of the tracheid and pit geometries (not dissimilar to our single-wood model in the absence of the effects of surface tension). The timber drying process results in a significant portion of the bordered pits becoming aspirated, for example Usta & Hale^40^ report that around 94% of pits were unaspirated for the green-wood spruce. Taking a proportion of unaspirated pits more appropriate for green-wood, our model then provides pit resistivities in the range $5\le {R}_{p}\le 9\,{\rm{MPa}}\,{\rm{s}}/{{\rm{mm}}}^{4}$, which agrees well with the measurements of Pittermann *et al*.^42^ — note that the dynamic viscosity of the chloroform used in our experiments is about half that of water. The only other values we are aware of, for the losses through bordered pits, are those of Lancashire & Ennos^27^ who reported the resistance from their laboratory analogue model of a single pit as being $1.7\times {10}^{15}\,{\rm{Pa}}\,{\rm{s}}/{{\rm{m}}}^{3}$. This value was noted by Pittermann *et al*.^42^ as being at the ‘low end’ of their empirical measurements; *cf*. our data yields reassuringly higher values for the resistance of a single pit, in the range $4\times {10}^{16}-6.5\times {10}^{16}\,{\rm{Pa}}\,{\rm{s}}/{{\rm{m}}}^{3}$.

While electrical resistivity (the inverse of conductivity) is an intrinsic material property, the same is not true for their counterparts, hydraulic resistivity and hydraulic conductivity, since these depend on the dynamic viscosity of the fluid flowing within the porous medium. However, the published literature (e.g. Pittermann *et al*.^42^) presents the losses due to the tracheid walls, and those due to the bordered pits via the hydraulic resistivity of each, namely ${R}_{w}=8\mu /\pi {r}_{T}^{4}$ and *R*_*p*_, respectively. The geometric resistance to flow of the timber pore space in our model, ${B}_{T}^{-1}$, is independent of the fluid properties and consists of two terms, one relating to the geometric resistance due to flow through the trachied tube bundle, and the other to the bordered pits — the ratio of these two terms is equivalent to assessing the ratio of hydraulic resistivity, i.e. $[3/({n}_{p}{a}_{p}^{3}{L}_{T})]/[8/(\pi {r}_{T}^{4})]\equiv {R}_{p}/{R}_{w}$. For green-wood stems belonging to the *Pinaceae* family, the data of Pittermann06 indicates that this ratio lies within the range $1.5\le {R}_{p}/{R}_{w}\le 15$. However, the agreement between experimental data and our model, combined with our micro CT measurements of the specimens, enables us to confidently assert that this ratio lies in the range $250\le {R}_{p}/{R}_{w}\le 10\,000$ for the softwood (Sitka spruce) timber specimens that we tested — the most significant variation of this ratio in our data being due to our model accounting for the difference between early- and late-wood phases with the volume averaged ratio falling in the range $3\,000\le {R}_{p}/{R}_{w}\le 5\,000$. This findings suggest that in dried softwood timber the resistance to flow is dominated by the bordered pits. When values representative of green-wood timber are taken for the proportion of unaspirated pits, our model suggests that this ratio lies within the range $100\le {R}_{p}/{R}_{w}\le 450$. The remaining difference between these values and those reported by Pittermann *et al*.^42^ can be accounted for by differences in the measurements of the tracheid lumen radius, with Pittermann *et al*.^42^ reporting ${r}_{T}\approx 6$ *μ*m for *Pinaceae* stems, whilst quantitative analysis of our CT measurements^34^ determined ${r}_{T}\approx 16$ *μ*m (such differences when raised to the fourth power introduce differences of factor 50, or so). Our findings suggest that in both green-wood and dried timber the resistance to fluid flow is dominated by the bordered pits.

### Scaling analysis

Examining the data in dimensionless form provides insight as to the leading order terms (for each model the leading order terms are the same). We examine the dimensionless intruded volume (scaled by pore space within the specimen) $\hat{V}(t)=V(t)/(\phi {A}_{0}{L}_{0})=2{L}_{f}(t)/{L}_{0}$, and time scales constructed from the dominant force balance between the retarding viscosity, *μ*, and a (driving) pressure scale. In our experiments only the imposed pressure, ${P}_{H}$, and the contribution from surface tension, Δ*P*_*c*_, are invariant with time, see (1). Initially we scale (9) and select the dimensionless time $\tau =t/(\mu /\Delta {P}_{c})$. Denoting $\Delta {P}_{a}={P}_{H}+{P}_{hyd}(t)-{P}_{in}(t)$ gives

14 $$\begin{array}{rcl}\hat{V}(\tau ) & = & 2{[\frac{2{B}_{T}}{\pi {r}_{T}^{2}{L}_{0}^{2}}{\int }_{0}^{\tau }\frac{\Delta P}{\Delta {P}_{c}}{\rm{d}}\tau ]}^{1/2}\\  & = & {[\frac{8{B}_{T}}{\pi {r}_{T}^{2}{L}_{0}^{2}}{\int }_{0}^{\tau }(\frac{\Delta {P}_{a}}{\Delta {P}_{c}}+1){\rm{d}}\tau ]}^{1/2}\\  & = & {[\frac{8{B}_{T}}{\pi {r}_{T}^{2}{L}_{0}^{2}}(\tau +{\int }_{0}^{\tau }\frac{\Delta {P}_{a}(\tau )}{\Delta {P}_{c}}{\rm{d}}\tau )]}^{1/2}.\end{array}$$

Knowing that $\hat{V}(\tau )\to 1$ for appropriate times $\tau \approx {\tau }_{c}$, Eq. (14) provides a means to evaluate the effects of surface tension on the intruded volume without need to evaluate the integral. Note that for larger times $\tau  > {\tau }_{c}$ the intruded fluid is in static equilibrium with $\Delta {P}_{a}=-\,\Delta {P}_{c}$ hence $\tau +{\int }_{0}^{\tau }\,\Delta {P}_{a}/\Delta {P}_{c}\,{\rm{d}}\tau =\tau +{\int }_{0}^{{\tau }_{c}}\,\Delta {P}_{a}(\tau )/$ $\Delta {P}_{c}\,{\rm{d}}\tau +{\int }_{{\tau }_{c}}^{\tau }-1{\rm{d}}\tau$ and (14) remains valid (and equal to unity). The two upper panes of Fig. 7 show that in our experiments static equilibrium is achieved at times ${t}_{c}\approx {10}^{3}\,{\rm{s}}$, giving $0.075 < ({\tau }_{c}\,8{B}_{T})/(\pi {r}_{T}^{2}{L}_{0}^{2}) < 0.11$ for our experiments. From this analysis we deduce that in our experiments the effects of surface tension, transport around 10% of the intruded fluid and the imposed pressure difference accounts for the remaining 90% of the transport. With this knowledge, it is then natural to use $\Delta {P}_{H}=\Delta {P}_{a}(0)={P}_{H}+{P}_{hyd}(0)-{P}_{in}(0)\approx {P}_{H}$ as the pressure scale in the analysis that follows.

We seek to analyse the intruded fluid volume by examining the variation in the dimensionless intruded volume $\hat{V}(\hat{t})=2{L}_{f}(\hat{t})/{L}_{0}$ with time $\hat{t}=t/T$, where $T\sim {L}_{0}/(d{L}_{f}/dt)$ is the characteristic filling time. From Eq. (7), this time scale is therefore $T\sim {L}_{0}\,\mu \,{r}_{T}^{2}\,{L}_{f}/({B}_{T}\,\Delta P)$. Using (8) to substitute for ${L}_{f}$, noting that to leading order ${\int }_{0}^{T}\,\Delta P\,{\rm{d}}t\sim T\,\Delta {P}_{H}$, and rearranging gives $T\sim \mu \,{L}_{0}^{2}\,{r}_{T}^{2}/(\Delta {P}_{H}\,{B}_{T})$. Knowing that ${a}_{p}\ll {r}_{T}$ and $\mathrm{8/3}\pi \approx 1$, Eq. (11) then gives ${B}_{T}^{-1}\sim {r}_{T}^{-4}+{({n}_{p}{a}_{p}^{3}{L}_{T})}^{-1}$, and so we define the characteristic filling time scale by

15 $$T=\frac{\mu \,{L}_{0}^{2}\,{r}_{T}^{2}}{\Delta {P}_{H}}(\frac{1}{{r}_{T}^{4}}+\frac{1}{{n}_{p}\,{a}_{p}^{3}\,{L}_{T}})=\frac{\mu \,{L}_{0}^{2}}{\Delta {P}_{H}\,{r}_{T}^{2}}\,(1+\frac{{r}_{T}^{4}}{{n}_{p}\,{a}_{p}^{3}\,{L}_{T}})\approx \frac{\mu \,{L}_{0}^{2}}{\Delta {P}_{H}\,{r}_{T}^{2}}\frac{{r}_{T}^{4}}{{n}_{p}\,{a}_{p}^{3}\,{L}_{T}}.$$

The data in this dimensionless form (lower panes of Fig. 7) exhibit a good collapse, indicating that the choice of time scale is appropriate. Physical insight into the liquid transport in softwoods is provided by considering *T* to be the time scale apparent on considering the dominant driving pressure to be forcing a viscous fluid to fill a tracheid tube bundle of length *L*_0_, multiplied by (one plus) the resistivity of the bordered pits relative to that of the tracheids. Note that for our data, the approximation in Eq. (15) introduces errors that were always less than 0.5%.

## Data Availability

All datasets generated and/or analysed during the current study are available from the corresponding author on reasonable request.
